# Colonic Tuft Cells: The Less-Recognized Therapeutic Targets in Inflammatory Bowel Disease and Colorectal Cancer

**DOI:** 10.3390/ijms25116209

**Published:** 2024-06-05

**Authors:** Ferenc Sipos, Györgyi Műzes

**Affiliations:** Immunology Division, Department of Internal Medicine and Hematology, Semmelweis University, 1088 Budapest, Hungary

**Keywords:** tuft cell, immune response, anti-inflammatory, anti-tumor, inflammatory bowel disease, colorectal cancer, therapy

## Abstract

Tuft cells are more than guardian chemosensory elements of the digestive tract. They produce a variety of immunological effector molecules in response to stimulation; moreover, they are essential for defense against protozoa and nematodes. Beyond the description of their characteristics, this review aims to elucidate the potential pathogenic and therapeutic roles of colonic tuft cells in inflammatory bowel disease and colorectal cancer, focusing on their primarily immunomodulatory action. Regarding inflammatory bowel disease, tuft cells are implicated in both maintaining the integrity of the intestinal epithelial barrier and in tissue repair and regeneration processes. In addition to maintaining intestinal homeostasis, they display complex immune-regulatory functions. During the development of colorectal cancer, tuft cells can promote the epithelial-to-mesenchymal transition, alter the gastrointestinal microenvironment, and modulate both the anti-tumor immune response and the tumor microenvironment. A wide variety of their biological functions can be targeted for anti-inflammatory or anti-tumor therapies; however, the adverse side effects of immunomodulatory actions must be strictly considered.

## 1. Introduction

Mucosal tissues constitute the most significant barrier between an organism and its environment. In the gastrointestinal tract, the cellular elements of the host organism are perpetually exposed to a complex luminal environment (the microbiome) composed primarily of nutrients and billions of commensals and occasionally pathogenic microorganisms. To maintain homeostasis, the host employs a complex tolerogenic or immunogenic response to differentiate between self- and non-self-antigenic structures. In the integration of the organismal response, the mucosal membrane at the interface between host tissues and luminal contents plays a prominent role [1,2]. The intestinal epithelium interacts with diverse trigger factors in the lumen to modulate the activation of the body’s most extensive immune apparatus, the gut-associated lymphoid tissue (GALT). The intestinal epithelium is a continually renewing structure; intestinal stem cells (ISCs) located at the base of the intestinal crypt provide a supply of differentiated cells dispersed throughout the epithelial lining. The integrity of the cell monolayer is required for barrier function to remain intact. Nonetheless, particular subtypes of epithelial cells, such as antimicrobial peptides produced by Paneth cells and mucus secreted by goblet cells, serve as additional protective factors. Recent studies have focused on an additional form of epithelial cells: the rarely occurring secretory epithelial tuft cells (TCs). Despite their discovery several decades earlier [3], the functional characterization of TCs was for a long time impossible in the absence of specific markers. Recent advances in the identification of TCs and the comprehension of signaling through luminal pathways, apical receptors, secondary messengers, and secretory mediators have enabled a more in-depth examination of TCs. It has become evident that these cells are additional determinants of mucosal immunity, as they serve as a link between the microbiome, nervous system, and immune system and also play a crucial role in the elimination of parasite and worm infections. The objective of this review is to discuss the morphological characteristics of TCs, their origin, the molecular basis of their differentiation, their identification, and their potential role in inflammatory bowel disease (IBD) and colorectal cancer (CRC) among the inflammatory background pathologies of the intestinal tract. In addition, we would like to highlight the potential therapeutic applications of TCs in IBD and CRC treatment.

## 2. The Structure of the Intestinal Mucosa

The intestinal mucosa consists of two primary components: the unilayered, cylinder-shaped epithelium and the lamina propria. The lamina propria, which is separated from the unilayered epithelium by a basement membrane, is composed of a variety of cells, including mesenchymal cells (fibroblasts, myofibroblasts, pericytes, endothelial, and smooth muscle cells) and immunocompetent cells (B- and T-lymphocytes, dendritic cells, and macrophages) [1,4]. Crypts and cilia are two spatially and functionally distinct compartments of the intestinal epithelium [5]. The stem cells are located at the base of the crypts and above them in the transit amplification zone [6]. Columnar stem cells expressing the leucine-rich repeat-containing G-protein-coupled receptor 5 (Lgr5) in the crypt are self-renewing and therefore capable of producing all differentiated cell types of the epithelium in vivo and in organoid cultures [7,8]. Paneth cells, which have undergone terminal differentiation, are co-localized with progenitor cells in the crypt’s basal region. The Paneth cell is the only differentiated, long-living intestinal epithelial cell that regulates the microbiome and serves as a niche for stem cells [9,10]. Three distinct categories of villous cells, namely, the most abundant nutrient-absorbing enterocytes, goblet cells, and enteroendocrine cells, have been recognized until recently [11]. The progeny cells derived from stem cells migrate to the upper portion of the crypt, where they differentiate into specific cell types prior to colonizing neighboring pads. In the region of Peyer’s plaques, progenitor cells differentiate into specialized antigen-presenting M cells (microfold cells) [12]. In addition, it has been known for a long time that the intestinal epithelium contains so-called TCs [12,13,14,15].

## 3. Morphological Characteristics and Origins of Tuft Cells

TCs can be found in tissues other than the alimentary tract, such as the airways, thymus, gallbladder, pancreas, and urethra [3]. The flask-shaped body of a TC has a narrow neck from which a “tuft” of microvilli extends from the apical membrane into the intestinal lumen [16]. TC microvilli are longer and more compact than those of adjacent cells and are connected to a network of apical–basal orientations rich in microfilaments and microtubules [17,18]. In TCs, microtubules extend deep into the nucleus and are attached to the perinuclear endoplasmic reticulum. Both at the base of the microvilli and between the microtubules, membrane-associated vesicles (so-called glycocalyx bodies) have been identified, suggesting a macromolecular exchange pathway between the intestinal lumen and the endoplasmic reticulum [16,17,18]. Intermediate filaments and even neurofilaments are also involved in the cytoskeletal architecture of TCs [19]. Some TCs also have laterally initiated cytoplasmic extensions, thus allowing them to reach the nucleus of neighboring cells and function as molecular transport pathways. These extensions of TCs extend from the apical junctional complex to the level of the nucleus and are often associated with desmosomes at their base [16,17]. In addition, their basal segments of extensions also form neuropod-like projections along the basal lamina, suggesting paracrine signal transduction [16,20,21], although secretory vesicles have not been observed within TC neuropods. In contrast, enteric neuronal fibers adjacent to TCs contain vesicles containing acetylcholine (ACh) for paracrine signaling [22].

**Figure 1:**
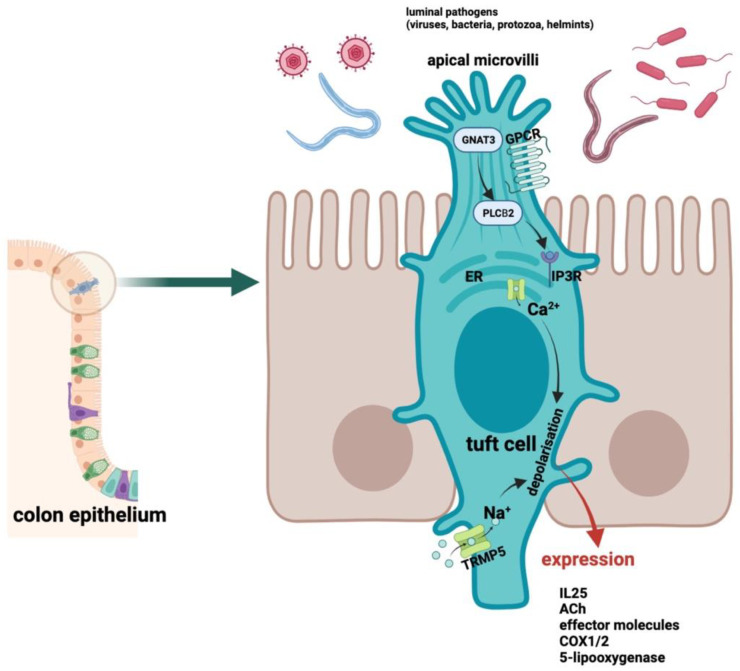
The structure and basic function of tuft cells. These cells detect and analyze luminal chemicals, including those that come from pathogens. The taste signal receptors of TCs are composed of heterotrimeric G-coupled guanine nucleotide-binding protein receptors. The activation of these unique receptors initiates a shared signal transduction pathway; it comprises GNAT3, PLCβ2, IP3R type 2, and TRPM5, which function as a monovalent-specific cation channel for Na^+^, K^+^, and Cs^+^ ions rather than Ca^2+^ ions. After depolarization of the cell membrane and activation of the receptors, the detected signal will undergo conversion. Thus, additional cells could detect the molecules that elicit a sufficient immune response. GNAT3: G Protein Subunit Alpha Transducin 3; GPCR: G protein-coupled receptor; PLCB2: Phospholipase C Beta 2; ER: endoplasmic reticulum; IP3R: Inositol trisphosphate receptor; TRPM5: Transient Receptor Potential Cation Channel Subfamily M Member 5; IL-25: interleukin 25; ACh: acethylcholine; COX: cyclooxygenase. This figure was partly created with www.BioRender.com (accessed on 28 April 2024).

It is not surprising, therefore, that TCs were first characterized by the expression of structural markers such as their apical brush-border-related villin protein [23], specific cytokeratins (e.g., CK18) [24], and their microtubule-associated proteins such as α-tubulin [25], although none of these are exclusive specificities. However, the recognition of chemosensory features shared by TCs and taste receptor cells has led to the discovery of new markers (α-gustducin, the TRPM5 cation channel) [26,27]. The detection of TRPM5 expression was a particularly major breakthrough, as it is now known to play a fundamental role in the functioning of TCs [28]. Microtubule-coupled protein kinase 1 (DCLK1) was originally reported to be a putative marker of intestinal epithelial stem cells but is predominantly expressed by TCs [29,30]. DCLK1 is widely used as a TC marker [25,31,32]. Figure 1 illustrates the structure and basic function of the tuft cells.

The intestinal epithelium has a particularly dynamic architecture, with highly proliferating Lgr5+ ISCs providing continuous renewal every 3–5 days [6,7,33]. Compared to other epithelial cells, intestinal TCs are quite rare and sporadically distributed; they constitute only 0.4–2.3% of the total epithelial population in the mouse intestinal epithelium [13,23,34], whereas the TC density of the human sigmoid colon is ~100 TC/mm^2^ [24]. As TCs exit the crypt and migrate along the axis of the pileus, their function, similar to that of enteroendocrine cells, can change during vigorous differentiation [25,26]. While the average renewal rate of TCs is 1–2 weeks, the average renewal rate of the TC subpopulation, which represents 5% of DCLK1+ TCs, is remarkably long, lasting up to 18 months [35]. These long-lived TCs may contribute fundamentally to epithelial regeneration following damage (of any origin) by maintaining their stem cell potential. Furthermore, TCs can also generate small and large intestinal organoids in vitro and may manifest as protumor cells upon the deletion of the adenomatous polyposis coli (APC) gene [36]. Meanwhile, it remains uncertain whether long-lived TCs represent a truly unique, specific cell population or whether they may correspond to a common secretory progenitor [28].

## 4. Differentiation of Tuft Cells

The identification of DCLK1 as a (mouse) TC marker has greatly facilitated its further characterization. It was found that the differentiation of ISCs into TCs is independent of the transcription factor neurogenin-3 (Neurog3), which, in turn, is essential for the formation of the enteroendocrine cell lineage; i.e., TC lineage is independent of enteroendocrine cell lineages [13]. Instead, the common differentiation marker for all TCs with different organ localizations is the taste-bud-cell-specific transcription factor Pou class 2 homebox 3 (Pou2f3). Pou2f3 is the master regulator and essential condition for the development of a TC; i.e., taste buds of the tongue and enteric TCs share a common cell lineage [36,37,38]. Yet, despite the morphological similarity between shared Pou2f3 and tissues, population-based and single-cell RNA sequencing transcriptional profiling studies have demonstrated a heterogeneity of TCs between and within tissues [23,29,30,39,40]. In the mouse small intestine, two TC progenitors and two mature TC clusters have been confirmed so far: one expressing the neural developmental gene signal tuftelin 1 (Tuft-1), while the other, Tuft-2, associates with genes regulating immunological functions [23]. Despite the overlapping expression patterns of Tuft-1 and Tuft-2, the secretion of thymic stromal lymphopoietin (TSLP), an epithelial-derived cytokine that prefers Th2 immune responses, is exclusively associated with Tuft-2, which, in turn, suggests functional and potential spatial heterogeneity of TCs [23,31]. The heterogeneity of TCs is also indicated by the region-specificity of another transcription factor associated with TC differentiation, AtonalbHLH (ATOH1), in the intestinal tract [23]. In the colon, ATOH1 is essential for differentiation into secretory cell lineages (goblet, Paneth, and enteroendocrine cells) and TCs, whereas small intestinal TCs are ATOH1-independent [32,36,41]. Krüppel-like factors (e.g., Klf3, Klf6) have also been identified as transcription factors of small intestinal TCs, whereas their roles in the development of colonic TCs are still unknown [23]. The regulator Sprouty2, which is mostly found in the colonic epithelium, is another intracellular signal transduction regulator that plays a role in TC differentiation [42]. Both in vitro and in vivo, it has been shown that Sprouty2 expression is reduced in patients with acute colitis, leading to TC and epithelial cell hyperplasia [42]. Studies conducted more recently have shown that inositol polyphosphate multikinase (IPMK), which controls cell growth and some immune functions, is also important for the development of TCs [43].

## 5. Identification of Human Intestinal Tuft Cells

There is no consensus yet on the identification and characterization of TCs in the human digestive tract. Although the presence of a DCLK1+ TC population in the human colon has been confirmed, there is also evidence that the morphology of human intestinal DCLK+ cells resembles that of absorptive enterocytes rather than the classical TC form [44,45]. Thus, it is questionable whether DCLK1 is indeed indicative of human TCs [44,45]. Differences in DCLK1 immunoreactivity may be due to differences in protocol and fixation methods, but it cannot be excluded that human TCs do not express DCLK1. In contrast, cyclooxygenase-1 (COX-1), the major enzyme regulating prostaglandin synthesis, is considered the most reliable marker of human TCs [13,24,46]. Other relevant TC markers include phosphorylated epidermal growth factor (p-EGFR), arachidonate 5-lipoxygenase (ALOX5), advillin (AVIL), girdin, and choline acetyltransferase (ChAT), although none of these are TC-specific [34,41,46,47,48,49]. As they are often restricted to TCs within the epithelial layer, the combination of any of the indicated markers with epithelial-cell-specific epithelial cell adhesion molecule (EpCAM) or E-Cadherin may be useful for the identification of TCs [46]. Table 1 lists the TC markers identified so far, indicating each marker’s TC specificity.

## 6. Tuft Cell Receptors, Chemical Sensing, and Signal Transduction Mediators

TCs use apical receptors to sense a wide range of changes in the intestinal lumen and are thus competent sentinels of the digestive system. They communicate with both the mucosal immune system and the neuronal network. The apical signaling of TCs is based on taste receptors similar to those in the taste buds of the tongue and soft palate. There are three types of receptors: the first detects signals from sweet and umami (the so-called fifth taste) substances, the second detects them from bitter substances, and the third detects them from sour substances [59]. TCs express type 1 (Tas1Rs) and type 2 (Tas2Rs) chemosensory receptors [60,61]. Another receptor is the succinate receptor 1 (SUCNR1), which senses the metabolite succinate secreted by certain bacteria, protozoa, and worms [57,62]. Succinate stimulates interleukin 25 (IL25) production through this receptor, thereby inducing a type 2 immune response, which is associated with the activation of type 2 naïve lymphoid cells (ILC2), eosinophilia, and increased TC and goblet cell differentiation [62]. The highest SUCNR1 expression has been observed in ileal TCs [63].

A tyrosine kinase receptor has also been identified in TCs, although its mechanism of activation is not yet clear. Other receptors with poorly understood functions, such as the gamma-aminobutyric acid (GABA) A/B receptors, the dopamine receptor Drd3, and the orphan adhesion G-protein receptor (ADGRG2), also contribute to basolateral signaling [23,26,57]. A pathway of IgG activation has also been reported in a small proportion of intestinal TCs (approximately 2.75% of mouse small intestines) [64]. The presumed immunomodulatory effect of GABA is protective, but there are also data on its deleterious functions [65,66]. The presence of the calcium-activated ion channel (TRPM5) is specific for intestinal epithelial TCs [31,38,59,67]. Although the associated mechanism is poorly understood, TCs also identify worms and protozoa through their taste receptors.

TCs are capable of synthesizing a myriad of molecules with paracrine and endocrine effector functions, including IL25, eicosanoids, acetylcholine, β-endorphin, and TSLP, all of which have unusual spectral functions [13,23,31,38,46,57].

IL25, produced by TCs, is considered a critical stimulated cytokine in the induction of type 2 immune responses against worms and protozoa [38]. The expansion of ILC2s ensures the early secretion of the key cytokines IL5, IL9, and IL13 [31,38]. IL13 induces intestinal epithelial remodeling, stimulates TC- and goblet-cell-directed differentiation of ISCs, and is of elementary importance in the anti-parasite immune response [31,38]. Parasite clearance is also promoted by IL4, IL5, and ACh produced by ILC2s [38,68]. Other immunocompetent cells of the lamina propria, such as NK-T cells and nuocytes (ILC2 effector cells), also play a role in the IL25-mediated anti-worm immune response [69,70]. 

The expression of COX-1/2 and 5-lipoxygenase allows TCs to synthesize PGD2 and PGE2, as well as cysteinyl leukotriene C4 (LTC4), molecules that play important physiological roles in both intestinal homeostasis and inflammation (e.g., IBD and colon cancer) [57,71,72]. Inflammation of the gut has been associated with increased COX-2 enzyme activity and PGE2 production [73,74], although PGE2 can exert both pro- and anti-inflammatory effects, depending on the context [75]. LTC4 is an additional inflammatory mediator, synthesized via the lipoxygenase pathway through a worm- and protozoan-stimulated, as-yet-unknown receptor mechanism [76,77,78]. Although LTC4 functions synergistically with IL25, its role in anti-worm immunity is unimportant, unlike that of IL25.

ACh, the major neurotransmitter of cholinergic neurons, is an important modulator of both epithelial proliferation and gut physiology [46]. In the human digestive tract, TCs are the only epithelial cells that express ChAT, an enzyme required for ACh production [46]. It is not yet clear exactly what stimulates ACh synthesis in TCs, but it is likely to be the result of the activation of the canonical taste receptor signaling pathway [79]. There are speculations that debris of bacterial origin may be able to stimulate ACh-dependent auto- and paracrine pathways, and these are certainly relevant for intestinal TCs [80].

In neurons, the vesicular acetylcholine transporter (VAChT) is responsible for the delivery of ACh to secretory organelles. In mice, only some TCs of the proximal colon express VAChT [81]. In the human context, VACh-immunoreactive epithelial cells have also been demonstrated in the distal colon; however, whether Ach can be released from TCs is still unclear [82]. 

The relevance of beta-endorphin and TSLP secretion by intestinal TCs is not yet clear [23]. All enteric TCs produce beta-endorphin, the secretion of which is dependent on the TRPM5 channel [13]. Part of basolateral TSLP secretion is derived from TCs, and its secretion is enhanced in the presence of tissue damage and pathogens [31]. TSLP is involved in the development of various immunological conditions, including IBD [83,84]. Table 1 summarizes the given markers’ characteristics, functions, and TC specificity.

## 7. The Role of Tuft Cells in IBD

Idiopathic inflammatory bowel diseases with a chronic course are characterized by recurrent flare-ups alternating with clinically asymptomatic periods accompanied by varying degrees of inflammation of the gastrointestinal tract [85]. The two most common entities are ulcerative colitis (UC) and Crohn’s disease (CD), whose pathomechanisms are still not fully understood [85]. Increasing evidence is supporting the notion that in CD, the levels of Th1 cytokines (e.g., tumor necrosis factor-α/TNFα/, interferon-γ/IFNγ/, IL12) and Th17 cytokines (e.g., IL17A, IL21, IL23) are significantly increased in the inflamed mucosa of afflicted patients, whereas in UC, the cytokine profile of the inflamed mucosa is associated with an overproduction of Th2 cytokines (such as IL4, IL5, IL13) [85].

Contrarily, significantly reduced IL25 levels were also detected in serum and intestinal mucosa samples from patients with exacerbated IBD, and a similar trend was observed in non-inflammatory mucosa and serum samples from patients with UC/CD in remission [86]. However, no distinction was made between epithelial and subepithelial IL25 expression in this study. Following infliximab treatment for IBD flare-ups, serum IL25 levels were normalized [86]. These findings suggest a role of IL25 in the pathogenesis of IBD; increasing IL25 levels may even be a potential therapeutic option.

**Figure 2:**
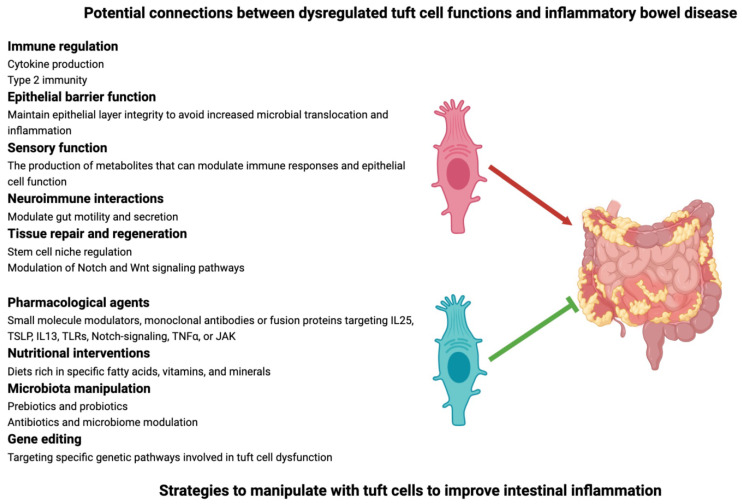
Role and therapeutic use of tuft cells in inflammatory bowel disease. The contribution of tuft cells to the pathogenesis of inflammatory bowel disease is becoming increasingly acknowledged. The figure’s upper portion illustrates a number of possible connections. Tuft cell modulation is a topic that is actively investigated in an effort to ameliorate inflammatory bowel disease. Several strategies, displayed in the lower part of the image, are being explored. The red arrow indicates that TCs contribute to disease progression, whereas the green arrow suggests that therapeutically targeted TCs may block disease progression. This figure was partly created on www.BioRender.com (accessed on 2 June 2024). Wnt: Wingless and Int-1; IL: interleukin; TSLP: thymic stromal lymphopoietin; TLR: Toll-like receptor; TNF: tumor necrosis factor; JAK: Janus kinase.

TCs are increasingly being recognized for their role in the pathogenesis of IBD (Figure 2). TCs play a role in maintaining the integrity of the intestinal epithelial barrier, which is often compromised in individuals with IBD [1]. The dysfunction of TCs could lead to increased permeability and inflammation. In human CD and TNF-induced ileitis in mice, the number of TCs in the inflamed ileum was reduced, similar to what was also shown in the non-inflamed colons of UC patients in remission [24,41]. Succinate administration in the experimental model reduced epithelial damage and inflammation, in parallel with provoking an increase in Th2 and ILC2 cell numbers and a decrease in Th17 and ILC3 cell numbers [41]. TCs express SUCNR1, suggesting active involvement of these cells, as supported by the fact that in Pou2f3-deficient and thus TC-deficient mice, the beneficial therapeutic effect of succinate was lost [41]. Succinate is physiologically derived primarily from parasites but may also be present in the commensal microbiome, reinforcing the role of TCs in the possible anti-IBD effects of the induced anti-parasite immune response [41].

TCs are implicated in tissue repair and regeneration processes in the intestine. Dysfunctional TCs may impair these processes, leading to the chronic inflammation and tissue damage characteristic of IBD [28]. In developing countries, UC is less common, while endemic gastrointestinal infections caused by parasites are much more frequent. According to the “hygiene hypothesis”, this phenomenon is a consequence of excessive hygiene and reduced tolerance to environmental antigens [87]. In mice, treatment with probiotic Trichuris suis ovae (TSO) increased the number of IL25-producing intestinal TCs [38]. Similarly, studies on human IBD have reported a possible beneficial clinical effect of TSO intervention, although the exact mechanism is still questionable [88,89,90]. In a DCLK1-knockout mouse UC model, significantly more severe morphological signs of intestinal inflammation were observed. Inflammation-induced proliferation and growth of ex vivo colonic organoids were reduced. Although the number of TCs was not substantially altered, the lack of DCLK1 expression resulted in impaired cell activation and, consequently, a reduced tissue repair response [91].

Some of the inositol polyphosphate multikinase (IPMK) single-nucleotide polymorphisms (SNPs) have been evaluated as risk factors for human IBD [92]. IPMK plays an important role in the maintenance of intestinal homeostasis. In an IPMK-depleted mouse model of colitis caused by dextran sulfate sodium (DSS), the number of TCs in the inflamed mucosa was found to be lower [43]. 

TCs can modulate the immune responses in the intestine by secreting cytokines and other signaling molecules [3]. The dysregulation of TC function may contribute to the aberrant immune responses seen in IBD. SH2-domain-containing inositol 5′-phosphatase (SHIP), also expressed by TCs, is a regulator of immune activation. In SHIP-deficient mice, CD-like inflammation involving the distal ileum develops [93]. SHIP protein levels and activity have also been found to be reduced in human CD [94]. SHIP-deficient TCs may be involved in mediating inflammation through increased tissue COX activity and PGD2/E2 production [95]. The reduced TC numbers and IL25 production associated with IBD may reflect an impaired immune regulatory status of the host, which clearly favors the development of certain pathologies [24]. However, as many immunocompetent cells of the lamina propria also secrete IL25, it is not certain that TCs alone are responsible for the observed changes [86]. It is also not yet clear whether the reduction in TC numbers in IBD is a consequence of the disease or a genuine pathogenic factor.

TCs are involved in sensing luminal contents, including pathogens and dietary factors [39]. Alterations in their sensory function may lead to inappropriate immune activation and inflammation in individuals with IBD. TCs can also communicate with nerves in the intestine, forming neuro-immune circuits that influence intestinal homeostasis [57,82,84]. Disruption of these interactions may contribute to IBD pathogenesis.

Emerging evidence suggests that TCs play a significant role in intestinal homeostasis and immune regulation, making them potential targets for therapeutic intervention in IBD.

## 8. Tuft-Cell-Based IBD Treatment Options and Their Controversies

Modulating TCs to ameliorate IBD is an area of active research, but it is still in the early stages (Figure 2).

Researchers are investigating ways of specifically targeting TCs to either enhance their protective functions or inhibit their pro-inflammatory effects. This may involve designing drugs that modulate TC signaling pathways or manipulate their secretory profiles. Research has shown that IL25 promotes TC proliferation and activation [96]. Therapies targeting IL25 or its receptors (e.g., indolepropionic acid) could potentially modulate TC activity and inflammation in IBD-afflicted individuals [97]. TSLP is another cytokine involved in TC activation and function [83]. Modulating TSLP signaling pathways (e.g., long-isoform TSLP blockade) may affect TC responses and inflammation in the intestine [98]. Nevertheless, similar to any therapeutic approach that targets the immune system, the inhibition of IL25 or TSLP may give rise to potential adverse effects. Hyporesponsive type 2 immune responses may result in heightened vulnerability to parasitic infections or compromised mechanisms for tissue repair [99]. Inhibiting IL25 or TSLP signaling may disrupt mucosal immunity, leading to alterations in the intestinal barrier, heightened susceptibility to microorganisms in the respiratory tract or intestines, or the exacerbation of IBD [100,101]. By inhibiting IL25 or TSLP signaling, allergic inflammation may be mitigated, which could have therapeutic applications for patients with allergic conditions [102,103]. Nevertheless, this approach may give rise to unforeseen repercussions, including the increased stimulation of alternative inflammatory pathways or the worsening of other immune-mediated disorders. The potential consequences of inhibiting IL25 or TSLP signaling include compromised antitumor immune mechanisms, which may result in diminished immunosurveillance and may facilitate tumor metastasis or progression [101,104,105]. The potential consequences of inhibition extend beyond the specific target tissues, affecting immune function systemically, contingent upon the mechanism of action. This has the potential to result in unintended consequences or off-target effects on immune cell populations and cytokine networks throughout the entirety of an organism [106].

IL13 is known to promote TC differentiation and activation [3]. Therapies targeting IL13 or its receptors (e.g., anrukinumab, tralokinumab, or pitrakinra) may indirectly influence TC function and contribute to IBD management [107,108]. In the case of IL13 inhibition, adverse effects may also manifest in various ways, including unintended immune-mediated conditions, off-target effects, and altered mucosal immune responses that compromise the immune barrier. Such consequences may include heightened vulnerability to respiratory infections and the exacerbation of inflammatory conditions, such as asthma. The suppression of protective immune responses through the inhibition of IL13 signaling may result in heightened vulnerability to respiratory infections, including bacterial and viral pneumonia [109]. However, some studies show that neither infections nor tumor events are significant after IL13 blockade [110]. IL13-targeted inhibitory therapies have the potential to hinder the mechanisms responsible for tissue repair, which could result in complications such as tissue fibrosis, delayed wound healing, or compromised pulmonary function [111].

Toll-like receptors play a role in sensing microbial stimuli and regulating TC responses [112]. Modulating TLR signaling with agonists or antagonists (e.g., cobitolimod, evitoran, or resatorvid) could potentially affect TC function and inflammation in IBD [113]. Notch signaling is involved in TC differentiation and maintenance [114]. Inhibitors of Notch signaling pathways (e.g., MRK-560) may alter TC populations and their responses in the intestine [114].

Although there is therapeutic potential in targeting TLR signaling for a range of inflammatory and autoimmune diseases, this approach may also result in specific adverse effects. Negative consequences induced by the deletion of particular TLRs (e.g., TLR4, TLR5, TLR9) or critical components of TLR signaling pathways underscore the significance of pattern recognition receptors in conditioning intestinal immune cells. Enhanced vulnerability to bacterial, viral, or fungal infections; the exacerbation of autoimmune disorders; the generation of systemic inflammatory responses (e.g., cytokine release syndrome); immunosuppression resulting in opportunistic infections or compromised anti-tumor immunity; tolerance; or hyporesponsiveness are among the potential adverse effects of TLR-signaling modulation [115,116,117,118,119,120,121].

Targeting Notch signaling may also result in specific adverse effects. Inhibition of Notch signaling may result in adverse effects such as dose-limiting gastrointestinal toxicity, hematopoietic abnormalities, hair loss, and neurological and cardiovascular complications [122,123,124,125,126,127,128]. The disruption of Notch signaling has the potential to impact the homeostasis and functionality of immune cells, resulting in immune dysregulation that manifests as modified T cell responses, compromised B cell development, or abnormal cytokine synthesis [129]. Moreover, depending on the tumor type and genetic background, inhibiting Notch signaling may have complex effects on tumorigenesis, potentially promoting or inhibiting tumor growth, metastasis, or drug resistance [123,130]. Notch signaling is crucial for organogenesis and embryonic development. Developmental or congenital malformations may result from the inhibition of Notch signaling during the course of development, including skeletal abnormalities, craniofacial defects, and organ malformations [131].

Further research is necessary to fully comprehend the effects of these molecules and pathways on TCs and develop safe and effective targeted therapies for IBD. Clinical trials evaluating these approaches are ongoing, and they may provide further insights into their therapeutic potential in the management of IBD.

Certain dietary components have also been shown to influence TC function [114,132]. Modulating one’s diet to promote the maintenance/development of a healthy TC population or to reduce inflammation may have therapeutic potential in IBD management. Dietary fiber serves as a substrate for fermentation by the gut microbiota, leading to the production of short-chain fatty acids (SCFAs) such as butyrate. SCFAs have been shown to promote TC differentiation and function, potentially enhancing intestinal barrier integrity and reducing inflammation in IBD [133]. Polyphenols, found in fruits, vegetables, and certain beverages like green tea, have anti-inflammatory and antioxidant properties. They may modulate gut microbiota composition and activity, indirectly influencing TC function and inflammation in IBD [134]. Omega-3 fatty acids, found in fatty fish, flaxseeds, and walnuts, have anti-inflammatory effects and may help reduce inflammation in IBD [135,136]. While their direct effects on TCs are not well studied, their overall impact on gut health may indirectly influence TC function.

SFCAs and polyphenols, however, may induce gastrointestinal disturbances, electrolyte imbalance, acidosis, obesity, and dyslipidemia. SCFAs possess anti-inflammatory properties and induce immune tolerance. However, an overabundance or disruption of SCFA synthesis may contribute to immune dysregulation, worsen inflammatory conditions (e.g., IBD) or allergic diseases, or disrupt barrier function, ultimately resulting in leaky gut syndrome [137,138].

Probiotics are live microorganisms that confer health benefits when consumed, while prebiotics are non-digestible fibers that promote the growth of beneficial gut bacteria. Both probiotics and prebiotics can modulate gut microbiota composition and activity, which, in turn, may influence TC function and inflammation in IBD [139]. Certain herbs and spices, such as curcumin, ginger, and garlic, possess anti-inflammatory properties. Incorporating these into one’s diet may help reduce inflammation associated with IBD and potentially modulate TC activity [139,140,141,142].

It is crucial to acknowledge that, although these nutritional interventions might offer potential advantages in the management of IBD, personal reactions may differ. Therefore, prior to implementing dietary modifications, individuals with IBD who may have particular dietary restrictions or sensitivities should consult with a registered dietitian or healthcare professional. Emerging technologies such as microbial engineering and synthetic biology allow for the manipulation of microbial communities to produce desired effects [143]. This approach holds promise for developing targeted interventions that directly impact TC function and inflammation in IBD. However, individual responses to microbial interventions can vary, highlighting the importance of personalized approaches in IBD management. Probiotics and prebiotics may also provoke adverse effects. Besides different gastrointestinal symptoms and allergic reactions, the potential aggravation of irritable bowel syndrome symptoms and the promotion of small-intestinal bacterial overgrowth syndrome have to be mentioned [144,145].

Biologic therapies that target important inflammatory pathways linked to IBD may change the overall inflammatory environment in the intestines, which could have an indirect effect on TC function. In regard to IBD, the development of specific biological therapies that target TCs directly is still in its nascent phase. However, several approved or currently researched biological therapies for IBD may indirectly impact the function of TCs or the associated inflammatory processes. TNFα plays a key role in the pathogenesis of IBD, serving as a pro-inflammatory cytokine. Anti-TNF agents, such as infliximab, target TNFα to reduce inflammation in IBD. While their direct effects on TCs are not well studied, they may modulate the inflammatory environment in the intestine, which could impact TC function [114]. Janus kinase (JAK) inhibitors, such as tofacitinib, inhibit signaling pathways involved in cytokine-mediated inflammation. Although specific effects on TCs have not been studied, JAK inhibitors modulate immune responses and reduce inflammation in IBD, potentially indirectly impacting TC biology [146].

While these biological therapies have demonstrated efficacy in treating IBD, their specific effects on TCs and their roles in disease management require further investigation. The side effect profiles of biological therapies are widely known. Among these, triggered autoimmunity, secondary infections, and malignancies must be highlighted [147,148]. 

With advances in gene editing technologies such as CRISPR-Cas9, it may be possible to directly manipulate TC genes to enhance their protective functions or inhibit their pro-inflammatory effects [149,150]. Gene editing specifically targeting TCs in IBD is still an area of ongoing research, and there are no direct gene editing results available targeting TCs in regard to IBD. When developing a gene editing method, researchers need to take into account undesirable on-target and off-target effects, the development of mosaicism, undesirable immune reactions against the Cas9 protein, and the emergence of undesirable long-term side effects. In addition, there are a number of ethical and regulatory aspects to consider. 

In sum, further research is necessary to fully comprehend the role of TCs in IBD pathogenesis and formulate safe and effective therapeutic strategies that target these cells. Clinical trials are underway to investigate some of these potential interventions, but it may take time before they are widely available for clinical use. However, when developing and administering different immunomodulatory treatments, it is always important to keep in mind the potential for serious and sometimes life-threatening side effects.

## 9. The Role of Tuft Cells in Colorectal Cancer

TCs may be involved in the process of tumorigenesis, although there are no data yet on their direct role in the development of CRC.

TCs can secrete inflammatory cytokines, such as IL-25, which can promote inflammation in the gastrointestinal tract [151]. Chronic inflammation is a known risk factor for CRC as it can lead to DNA damage and mutations in cells, promoting tumorigenesis [152]. The number of DCLK1+ TCs has been significantly increased in several models of inflammation-induced carcinogenesis, suggesting a potential role in malignant transformation [14,153,154]. In a Dclk1-CreERT2 knock-in mouse model, DCLK1+ cells were short-lived and rarely functioned as intestinal stem cells under physiological and pathological conditions, but they did function as cancer stem cells in tumors formed in ApcMin/+ mice [35]. To further investigate the DCLK1 lineage in relation to homeostasis, regeneration, and carcinogenesis, Dclk1-CreERT BAC transgenic mice were developed, which, unlike the previously reported Dclk1-CreERT2 knock-in mice, contained two intact endogenous Dclk1 loci. The Dclk1-CreERT BAC transgenic mouse model faithfully marks intestinal villus cells and identifies a small subset of DCLK1+ cells that are exceptionally long-living and quiescent [27]. These cells require neuronal input for survival and are involved in the epithelial response to injury. More importantly, these DCLK1+ cells remain quiescent and long-lived for a long time, even in the presence of APC loss, but become potent cancer-initiating cells upon the induction of inflammation [27].

In the human colonic mucosa, COX-2+ TCs have been identified, and they are located in the epithelium in association with subepithelial COX-1- and COX-2-expressing cells (probably pericryptal fibroblasts) near the stem cell zone [155]. Rare pericryptal fibroblasts may stimulate the expression of regeneration-related stem cell markers (e.g., Sca-1+ cells) and thus have tumor-initiating effects through paracrine PGE2 signaling [155,156]. TCs are the major source of prostaglandins in the intestinal tract. Both imbalances in arachidonic acid metabolism (including prostaglandin synthesis) and the production of free oxygen radicals play a central role in the pathogenesis of colorectal cancer. These findings suggest that a role of TCs in the development of CRC cannot be excluded [13].

TCs can influence the immune response in the gut by interacting with immune cells [39,84,157]. This modulation of the immune system can create an environment favorable for tumor growth and progression by suppressing anti-tumor immune responses and promoting immune evasion by cancer cells [152].

TCs may induce epithelial–mesenchymal transition (EMT) in neighboring cells, a process where epithelial cells acquire mesenchymal properties. By conducting a series of carefully planned experiments using mouse models of pancreatic cancer and pancreatic cancer cell lines, our understanding of the role of Dclk1 in cancer has been significantly expanded. Dclk1 expression is elevated in human pancreatic cancer samples and early murine pancreatic intraepithelial neoplasias. Moreover, the downregulation of Dclk1 via siRNA results in the upregulation of miRNA200a, which harms EMT [158]. Analogous to the findings observed in CRC, the absence of Dclk1 resulted in an increase in let-7a expression and a decrease in c-myc expression [158,159]. This finding indicates that Dclk1 may be involved in the progression of cancer, as c-myc is a critical element in the development of pancreatic and colorectal malignancies [160,161]. Altered EMT can enhance cell motility and invasiveness, enabling cancer cells to invade surrounding tissues and metastasize.

TCs can modify the microenvironment of the gut, influencing factors such as nutrient availability, oxygen levels, and signaling molecules [3,162,163,164]. These alterations can create conditions favorable for cancer cell survival, proliferation, and invasion [165].

The 11q23.1 inherited genetic variation is associated with an increased risk of colorectal cancer. Based on weighted gene co-occurrence network analysis (WGCNA), it is hypothesized that 11q23.1 trans-eQTL targets (Expression Quantitative Trait Loci) form a Pou2Af2-associated network that is likely to be tuft-cell-specific, and reduced expression of these genes in silico correlates with reduced TC frequency [134,166]. 

In general, TCs influence the microenvironment of the intestine, promote inflammation, modulate the immune response, and induce EMT, all of which contribute to the initiation of CRC.

## 10. Tuft-Cell-Based CRC Therapies and Their Considerations

**Figure 3:**
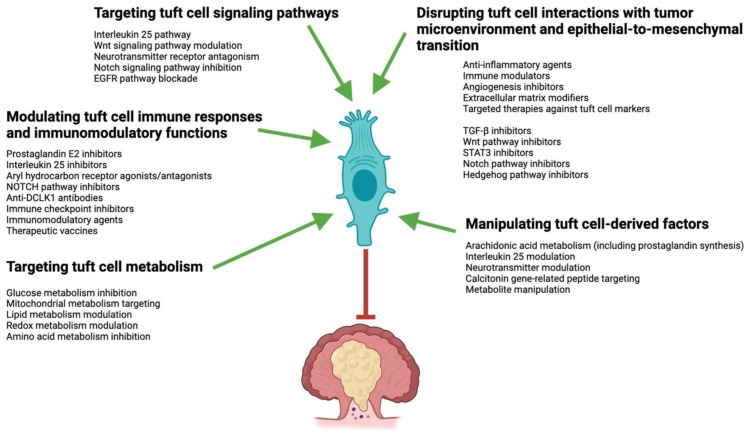
Tuft cells serving as potential therapeutic targets in colorectal cancer. These are the potential ways in which the anti-tumor effects of tuft cells might be harnessed for therapeutic applications. The image displays potential treatment targets in conjunction with current therapy choices. The figure was partly created on www.BioRender.com (accessed on 28 April 2024).

TCs play a crucial role in tumor progression and immune regulation, making them potential targets for CRC therapy (Figure 3). Some compounds and molecules being investigated for targeting TC signaling pathways in CRC include PGE2 inhibitors, IL25 inhibitors, aryl hydrocarbon receptor (AHR) agonists and antagonists, Notch pathway inhibitors, and antibodies targeting TC markers [112,114,167,168,169,170,171,172,173,174]. TCs produce PGE2, which promotes tumor growth and immune evasion. Inhibitors of PGE2 synthesis or signaling pathways (e.g., N-[(1S,3S)-3-carbamoylcyclohexyl]-1-(6-methyl-3-phenylquinolin-2-yl)piperidine-4-carboxamide) may help counteract the immunomodulating effects of TCs in CRC [112,167]. PGE2 inhibition has long been extensively utilized to treat inflammatory conditions. The profiles of gastrointestinal, renal, cardiovascular, hepatic, hematological, neurological, respiratory, and allergic adverse side effects are well known and must be considered [175]. TCs also produce IL25, which contributes to the inflammatory microenvironment in individuals with CRC. Inhibiting IL25 signaling could hinder TC-mediated tumor promotion. Antibodies targeting IL25 may help in neutralizing its immunosuppressive effects mediated by TCs, potentially enhancing anti-tumor immune responses [168,169,170]. The TC/ILC2 axis, which connects homeostasis and defense in the gut, is affected by AHR signaling [171,172]. Modulating AHR activity with agonists or antagonists (e.g., AhR ligands, such as dietary- and intestinal-microbiota-derived compounds) may impact TC-mediated processes in CRC. However, AHR inhibition can also have serious side effects, such as immunosuppression, impaired metabolism of xenobiotics, endocrine dysfunction, neurotoxicity, or even pro-tumor effects [176,177]. TCs express Notch receptors and their ligands, which are involved in TC differentiation and function [114]. Inhibiting Notch signaling (e.g., MRK-560) could potentially disrupt TC activities in CRC [112,114]. Additionally, antibodies against specific TC markers, such as DCLK1 or POU2F3, are being explored for their potential to selectively target and eliminate TCs in CRC [168,173,174].

TCs can influence the immune response in the gut by interacting with immune cells. This modulation of the immune system can create an environment favorable for tumor growth and progression by suppressing anti-tumor immune responses and promoting immune evasion by cancer cells. Several drugs, compounds, and molecules are being investigated for targeting the immune modulatory effects of TCs in CRC. Besides anti-IL-25 antibodies and PGE2 inhibitors, these include immune checkpoint inhibitors, therapeutic vaccines, and immunomodulatory agents [173,174].

Immune checkpoint inhibitors (ICIs) targeting molecules such as PD-1, PD-L1, and CTLA-4 are being investigated to enhance anti-tumor immune responses in relation to CRC. Through IL13 and PDG2 secretion, and the DCLK1/Hippo interaction, TCs may influence the expression of these checkpoint molecules in the tumor microenvironment, and targeting them could help overcome TC-mediated immunomodulation [173,178]. Moreover, cell-based targeting of tumor-associated antigens or specific TC markers may help in activating and enhancing anti-tumor immune responses in CRC. By targeting TCs or antigens associated with TC activity, these modalities aim to stimulate the immune system to recognize and eliminate tumor cells. Nevertheless, ICIs can also give rise to other immune-related adverse events, such as dermatologic, gastrointestinal, endocrine, pulmonary, rheumatologic, neurologic, cardiovascular, or ophthalmic complications [179]. These adverse events have the potential to necessitate the permanent cessation of therapy. 

Several therapeutic medicines that precisely target DCLK1 have been created, including small-molecule kinase inhibitors such as LRRK2-IN-1, XMD8–92, DCLK1-IN-1, and XMD-17–51 [180,181,182,183]. Furthermore, there exist nanoparticle-encapsulated siRNA, monoclonal antibody CBT-15, monoclonal antibody and nanoparticle conjugate DCLK1-HA-Peg-plga, along with the chimeric antigen receptor T cells (CAR-T) CBT-511 cells, which are derived from DCLK1 monoclonal antibodies and DCLK1-modified using CRISPR/Cas9 [184,185,186,187,188]. Previous research has demonstrated that the LRRK2-IN-1 kinase inhibitor has the capacity to hinder the growth, movement, and infiltration of colorectal cancer cells. Moreover, the application of CAR-T therapy to selectively target colorectal cancer stem cells suggests that DCLK1 has promise as an immunotherapeutic target [188]. The newly developed DCLK1-IN-1 has shown the capability to avoid unintended consequences as a result of its exceptional pharmacokinetics [189]. Recent research has provided data supporting the efficacy of DCLK1-IN-1 in inhibiting the stem cell-like characteristics of CRC. This leads to a decrease in the development of primary and secondary tumors in living beings without causing significant harm to the liver and kidneys [173,189].

Increasing evidence supports the important role of DCLK1 in regulating tumor immunity, making it a promising target for therapeutic approaches against solid tumors. CRC patients who exhibited resistance to 5-fluorouracil (5-FU) treatment showed a significant elevation in DCLK1 levels. This indicates a potential correlation between DCLK1 and resistance to 5-FU in CRC [190]. DCLK1-IN-1, a chemical, has demonstrated the capacity to selectively target DCLK1 and successfully suppress CRC stemness. This inhibition was accomplished by interrupting the CCAR1/β-catenin pathway, leading to a decrease in resistance to the chemotherapeutic medication 5-FU [154].

The development of DCLK1 inhibitors that can be safely used in humans is still awaited [182,190]. There are a number of possible side effects (e.g., gastrointestinal and hepatological disturbances, cardiovascular, neurological, renal, and endocrine adverse effects, immunosuppression) that could affect the success of this new type of treatment.

Various immunomodulatory agents, such as toll-like receptor agonists, cytokines, and immune cell modulators, are being investigated in CRC to enhance anti-tumor immune responses [191,192]. These agents may target pathways affected by TC-mediated immunomodulation, promoting a more favorable immune microenvironment for anti-tumor activity.

Targeting the effects of TCs on EMT in CRC is an area of active research. Besides Notch pathway inhibitors, some drugs, compounds, and molecules being investigated for this purpose include inhibitors of the TGFβ, Wnt, STAT3, and Hedgehog pathways [193,194,195,196,197,198].

TCs can produce TGFβ, which promotes EMT and contributes to tumor progression in CRC. Inhibitors of TGFβ signaling pathways (e.g., fresolimumab, SB431542, SB505124, LY2157299, LY550410, AP12009, Lucanix, GC1008, or CAT-192) may help in blocking the effects of TC-derived TGFβ on EMT, potentially suppressing tumor metastasis and invasion [193,194]. Activation of the Wnt pathway by TCs can stimulate EMT and drive tumor progression in CRC. Some drugs that target parts of the Wnt signaling pathway might help stop the effects that TC has on EMT and tumor metastasis [193]. Signal transducer and activator of transcription 3 (STAT3) signaling is involved in TC function and EMT regulation in CRC. Inhibitors of STAT3 signaling pathways (e.g., STX-0119, N4, C188-9, L61H46, or AZD9150) may help in disrupting TC-mediated effects on EMT, potentially inhibiting tumor metastasis and enhancing sensitivity to chemotherapy [195,196]. On the other hand, it should not be forgotten that teratogenicity, immunosuppression, autoimmune reactions, and hematological abnormalities may result from the inhibition of TGFβ or STAT3 [199,200]. Inhibition of TGFβ may additionally result in compromised tissue remodeling, mucosal inflammation, and impaired wound healing [199]. STAT3 signaling inhibition may result in adverse neurological effects, including cognitive impairment, mood disorders, and neuropathies, by interfering with neurological function [200].

Hedgehog signaling components expressed by TCs can influence EMT and tumor progression in CRC. Inhibitors targeting the Hedgehog pathway (e.g., vismodegib, sonidegib, or glasdegib) may help in blocking TC-associated effects on EMT and metastasis [197,198].

TCs have been implicated in promoting angiogenesis, i.e., the formation of new blood vessels within the tumor microenvironment. Inhibitors targeting angiogenic pathways, such as vascular endothelial growth factor and angiopoietin signaling, may help in inhibiting tumor angiogenesis and disrupting the pro-tumorigenic effects of TCs [101,201]. Additionally, TCs can influence the composition and remodeling of the extracellular matrix (ECM) in the tumor microenvironment, which can impact tumor growth and metastasis. Agents targeting ECM components or enzymes involved in ECM remodeling may help in modifying the tumor microenvironment and impairing TC-mediated effects on tumor progression [202,203].

Currently, the investigation of pharmaceuticals, compounds, and molecules targeting TC signaling pathways in relation to colorectal cancer is limited to the aforementioned examples. Ongoing research endeavors to identify and assess novel therapeutic approaches that target the disruption of tuft-cell-mediated effects on colorectal cancer tumor progression and immune response.

## 11. Conclusions

Overall, our knowledge of TCs is still limited, especially with regard to their involvement in human diseases. Observations from studies using mice suggest that TCs are involved in a number of diseases, such as CRC and IBD. However, there are still few studies on human TCs, with one reason being that the relevant markers have only recently been identified. These are still being investigated; however, possible markers, although not specific to human TCs, include COX-1, p-EGFR, SOX9, ALOX5, AVIL, girdin, and ChAT [3].

Further research is required to clarify the molecular mechanisms and signaling pathways through which intestinal tuft cells impact immune responses and gastrointestinal homeostasis in humans. There is a need for research into the function of tuft cells in infectious and non-infectious gastrointestinal disorders. Furthermore, the potential role of TCs in gut microbiota regulation requires further investigation. It is critical to identify TC-specific markers in order to facilitate their isolation and investigation. Furthermore, it is necessary to develop and improve methodologies that enable the visualization and quantification of TCs in tissue samples. It is essential to conduct additional research on the genetic and epigenetic factors that govern the differentiation and function of tuft cells. This must include an examination of how environmental factors influence TCs’ biology. By focusing on these research avenues and procedures for clinical application, we can efficiently implement the insights from studies on intestinal TCs to improve prognosis, therapy, and administration in relation to gastrointestinal disorders.

The number of TCs in the colon appears to be altered in human disease settings. Whether this is merely a consequence of the disease or an actual pathogenic factor is a question that requires further investigation. The stimulation of TCs with other colonic epithelial cells or infectious agents could theoretically form the basis for the development of new types of therapies for promoting epithelial regeneration [96]. In addition to their chemosensory function, TCs also exert multiple immunomodulatory effects. Targeted and even combined blockade of these functions could form the basis for a large number of anti-inflammatory and anti-tumor therapies. However, we must recognize that the specific adverse effects of various tuft-cell-related immunomodulatory targeted therapies can vary depending on the specific context, the underlying disease, and the inhibition mode used. To fully understand the safety profile and possible side effects of novel treatment options, more research and clinical trials will need to be performed.

## Figures and Tables

**Table 1:** Markers for the identification of intestinal tuft cells.

Markers’ Main Functions	Marker	Immunoreactivity on Other Intestinal Cells (TC-Specificity)
**Structural**		
	Villin-1	also appears in other intestinal epithelial cells, but the basal staining in this case is tuft-cell-specific [50]
	CK18 (Cytokeratin-18)	also appears on some secretory cells [51]
**Chemical sensing**		
	TRPM5 (Transient Receptor Potential Cation Channel Subfamily M Member 5)	also appears on enteroendocrine cells [52]
	PLCβ2 (Phospholipase C β2)	also appears on enteroendocrine cells [52]
	SUCNR1 (Succinate Receptor 1)	not known [53]
	GNAT3 (G Protein Subunit Alpha Transducin 3)	also appears on some enteroendocrine cells [54]
**Neuronal**		
	DCLK1 (Doublecortin Like Kinase 1)	also appears on some enteroendocrine cells [36,55]
	ChAT (Choline acetyltransferase)	not known [56]
**Immunological**		
	COX-1/2 (Cyclooxygenase-1/2)	not known [13,57]
	HPGDS (Hematopoietic Prostaglandin D Synthase)	not known [13]
	ALOX5 (Arachidonate 5-lipoxygenase)	not known [57]
	IL25 (Interleukin 25)	not known [31,38]
	SiglecF (Sialic Acid Binding Ig-like Lectin F)	not known [38]
	PTPRC (Protein Tyrosine Phosphatase Receptor Type C)	not known [23,58]
	SH2D6 (SH2 Domain Containing 6)	not known [23,58]
**Transcription factor**		
	Pou2f3 (POU class 2 homeobox 3)	not known [38]
	Gfi1B (Growth Factor Independent 1B Transcriptional Repressor)	not known [36,38]
	Spib (SBIP transcription factor)	not known [58]
	SOX-9 (SRY-box transcription factor 9)	it also appears on Paneth and intestinal stem cells [38]

## Data Availability

No new data were created.
